# Feeding potential of adult *Systena frontalis* (Coleoptera: Chrysomelidae) on leaves of *Hydrangea paniculata* (Cornales: Hydrangeaceae)

**DOI:** 10.1093/jisesa/iead076

**Published:** 2023-08-26

**Authors:** Eleanor L Lane, Alejandro I Del Pozo-Valdivia

**Affiliations:** Department of Entomology, Hampton Roads Agricultural Research and Extension Center, Virginia Polytechnic Institute and State University, Virginia Beach, VA 23455, USA; Department of Entomology, Hampton Roads Agricultural Research and Extension Center, Virginia Polytechnic Institute and State University, Virginia Beach, VA 23455, USA

**Keywords:** choice assay, defoliation, feeding behavior, nonchoice assay, ornamental

## Abstract

*Systena frontalis* (F.) (Coleoptera: Chrysomelidae), also known as the red-headed flea beetle, is a defoliating pest of a variety of crop systems, such as ornamentals and food crops. Leaf consumption by this beetle renders ornamental nursery plants, such as hydrangeas (*Hydrangea paniculata* Siebold, Hydrangeaceae), unsaleable. In Virginia, this insect has become a major pest at commercial nurseries, and their feeding potential on affected crops has not been quantified. In this study, the extent of their damage to individual leaves and host preference between leaf ages were determined. The rate of defoliation on mature and young hydrangea leaves was measured over 24 and 48 h and between different numbers of adults. A single adult caused up to 10% damage to a young leaf or 5% to a whole mature leaf in 24 h. Without choice, there was a higher percent damage to young leaves. When the size of leaves was controlled by cut-out mature leaves, the area damaged was still higher in young leaves when compared with mature leaves. Adult feeding between mature or young leaves was further investigated by choice assays on a caged plant and within a containerized system. In these choice assays, adults inflicted higher percent damage on mature leaves in both caged plant assays and containerized direct choice assays. The choice assays were more similar to field conditions than the nonchoice assays. This demonstrates that *S. frontalis* showed a preference for mature leaves over young leaves within hydrangeas.

## Introduction

*Systena frontalis* (F.) (Coleoptera: Chrysomelidae), commonly referred to as the red-headed flea beetle, is a defoliating pest of ornamentals in nurseries (Lane and Del Pozo-Valdivia 2021, 2023) as well as other crop systems such as soybeans (NDSU 2022) and cranberries (Jaffe et al. 2021). The presence of this pest is distributed across the eastern United States, including Virginia (Joseph et al. 2021). Mated females insert their eggs in the potting media of nursery containers. After eclosion, larvae will feed on root hairs. Pupae will remain inside the pots, and then adults will emerge from the potting media (Lane and Del Pozo-Valdivia 2021, 2023, Lane 2022). Adult feeding results in leaves with shot holes and skeletonization that are unsightly and ornamentals that are unsaleable (Lane and Del Pozo-Valdivia 2021, 2023). As a polyphagous insect, they feed on a wide variety of economically important nursery crops (Joseph et al. 2021). The threat they pose to nurseries in eastern Virginia is clear and the extent has not been fully quantified. Adult density, in relation to defoliation, has been studied in cranberries and hydrangeas. Higher numbers of *S. frontalis* will cause more damage in a positive linear fashion on hydrangeas (Lane 2022), and cranberries (Jaffe et al. 2021).

Feeding preferences of *S. frontalis* have not been determined on hydrangeas (*Hydrangea paniculata* Siebold, Hydrangeaceae) or other ornamentals. Injury to cranberries is inflicted on leaves closer to the apical end of uprights (younger growth) and less so the further away from the apical end (older growth) (Jaffe et al. 2021). While this has been shown in a laboratory setting, choice assays, as well as percent defoliation, among leaf ages (young vs. mature) have not been quantified. Polyphagous lepidopterans such as some geometrids and erebids prefer mature leaves when given a choice (Cates 1980), and this concept may apply to *S. frontalis* since they are also polyphagous. Beetles within the same family as *S. frontalis* (Chrysomelidae) have been found to feed on young leaves in choice assays (Ernest 1989).

There are several methods of quantifying insect herbivory, including visual inspection, surface area meter, and picture-based software for leaf area. LeafByte is a mobile application that has been utilized in lieu of other more complex and time-consuming software (Getman-Pickering et al. 2020). It can be used in the field since it has a user-friendly interface and maintains a high level of accuracy compared with other methods of measuring herbivory (Getman-Pickering et al. 2020). Some of the examples of using this software include measuring herbivory for Science, Technology, Engineering and Mathematics (STEM) students in field trips during the Coronavirus disease (COVID-19) pandemic (Berke and Clark 2021), as well as measuring leaf area for insecticide bioassays for the Colorado potato beetles (Bažok et al. 2021). Based on the merits and applicability of this mobile-based method, it was used in this study to supplement the measurements taken by a surface area meter. Visual assessment of percent defoliation is one of the methods to measure herbivory that requires no instrumentation or removal of damaged tissue (Johnson et al. 2016). It is accurate and precise when performed by trained individuals (Johnson et al. 2016). Visual assessment for herbivory quantification benefits from using standardized references and scales. For instance, the reference for percent defoliation in hydrangeas proposed by Chong (2021) could be an accurate method to be used in entire plant injury estimations.

Investigating the feeding potential will provide insight into the biology of adult *S. frontalis*. The objectives of this study were (i) determining feeding preference between leaf ages in a nonchoice and whole-plant setting; (ii) documenting feeding potential within a Petri dish nonchoice assay based on leaf age, number of adults, and time of exposure; and (iii) determining feeding preference in laboratory choice assays based on leaf age and adult sex.

## Materials and Methods

### Individual Leaf Petri Dish Nonchoice Assays

Adult *S. frontalis* were vacuum-collected from hydrangeas var. ‘Limelight’, grown under open-field conditions in a gravel pad at the Hampton Roads Agricultural Research and Extension Center (HRAREC) in Virginia Beach, VA, up to 4 times between May and August 2021 and 2022. After each collection from the pad, adults were placed in a ventilated bucket temporarily for transportation to the laboratory. Two circular pieces of coffee filter paper moistened once with 1 ml of distilled water, lined 100-mm Petri dishes for hydration (Ernest 1989). Four dishes (replicates) of each treatment were prepared for 1, 3, and 5 adults at each 24- and 48-h exposure time. The adults were kept in a growth chamber (Percival, model I-30BLL, Perry, IA) for 24 h to ensure controlled conditions while starving. The growth chamber was set to 26 °C, with 55 ± 5% relative humidity (RH) and a 14:10 light:dark (L:D) regime. The next day, 24 new growth hydrangea leaves var. ‘Limelight’ and 24 older growth leaves of a similar size with no damage were collected from the gravel pad at the HRAREC. An additional 24 older growth leaves were collected and 9 cm^2^ squares were cut out to be used in comparisons of experimental area to standardize the sizes between leaf types (age of the leaf: young vs. mature).

The surface area of the undamaged leaves was measured 3 times using a LI-COR LI-3100C area meter (LI-COR Environmental, Lincoln, NE). The leaves were placed in their respective Petri dishes, and the dishes were wrapped in parafilm to prevent desiccation. At the end of the respective time periods (24 and 48 h), the adults were suctioned out of the Petri dishes and the leaves were removed for analysis. The injured areas of the leaf that had not been entirely eaten through were removed using forceps under a stereoscope. The surface areas were measured again three times using the LI-3100C area meter and the LeafByte mobile application (Zoe and Abigail Getman-Pickering, Ithaca, NY; Getman-Pickering et al. 2020). Multiple measurements aided in corroborating the leaf area and also increased precision. Individual measurements were then averaged and assigned to their respective experimental units. Assays with whole leaves, both mature and young growth, were repeated 4 times, and the ones with square mature leaves were repeated twice.

Data were analyzed using linear mixed models (PROC MIXED; SAS v9.4, Cary, NC). The response variables were both percent defoliation calculated from the LiCor and from LeafByte, as well as area consumed (cm^2^). Fixed effects were leaf type, number of adults, length of exposure, and their respective interactions. Replication (*r* = 4) was nested within each repetition and was included as a random effect. Two separate analyses were run with this data set. The first analysis included response variables collected from both young and mature whole leaves. The second one only compared variables between young whole leaves and mature 9 cm^2^ cut-out leaf squares, as an attempt to provide similar leaf areas to experimental adults. Degrees of freedom were calculated using the Kenward–Roger correction method (Kenward and Roger 1997). Mean separation post-analysis of variance (ANOVA) was calculated by using Tukey’s honestly significant difference (HSD) at α = 0.05. Percent defoliation on hydrangea leaves and leaf area consumed by adults were arcsine-square root and square root transformed, respectively, to meet the assumptions of homogeneity of variance and normality for these analyses. Then, percent defoliation and area consumed data were back-transformed, averaged, and presented as mean ± standard error.

### Old Versus New Growth Whole-Plant Choice Assays

Four 1-yr-old and nonflowering hydrangea plants var. ‘Limelight’ were chosen from the HRAREC for this experiment. These plants were in 11.4-liter pot and grown in pine bark potting media. Unsexed adult *S. frontalis* were collected randomly from infested hydrangeas at the HRAREC on 6 June, 18 August, and 1 September 2022, and 25 were placed in each 60 × 60 × 91 cm mesh cage (RestCloud, Zhejiang, China), with one hydrangea per cage. The caged plants with adults were left for 7 days in a plastic greenhouse (temperature between 30 and 35 °C, RH ~65%, and a 14:10 L:D regime) with irrigation and fertilization following commercial standards. After adults were removed, 10 young leaves and 20 mature leaves were chosen from across all layers of the plant to be assessed. The ratio of young to mature leaves selected in this experiment was a representation of an average ratio for a 1-yr-old hydrangea plant. The percent defoliation for each leaf was visually estimated using the Clemson University guidelines (Chong 2021). This assay was repeated 3 times in 2022. Percent defoliation at the leaf level was averaged within each leaf type (young vs. mature). These averages were arcsine squared-root transformed and then analyzed using a linear mixed model (PROC MIXED; SAS). The model included leaf type, repetition, and their interaction as fixed factors. Replication (*r* = 4) was the only random factor. Degrees of freedom calculation and post-ANOVA mean separation followed the same procedures as described in the previous section.

### Laboratory-Containerized Choice Assays

**Fig. 1.**
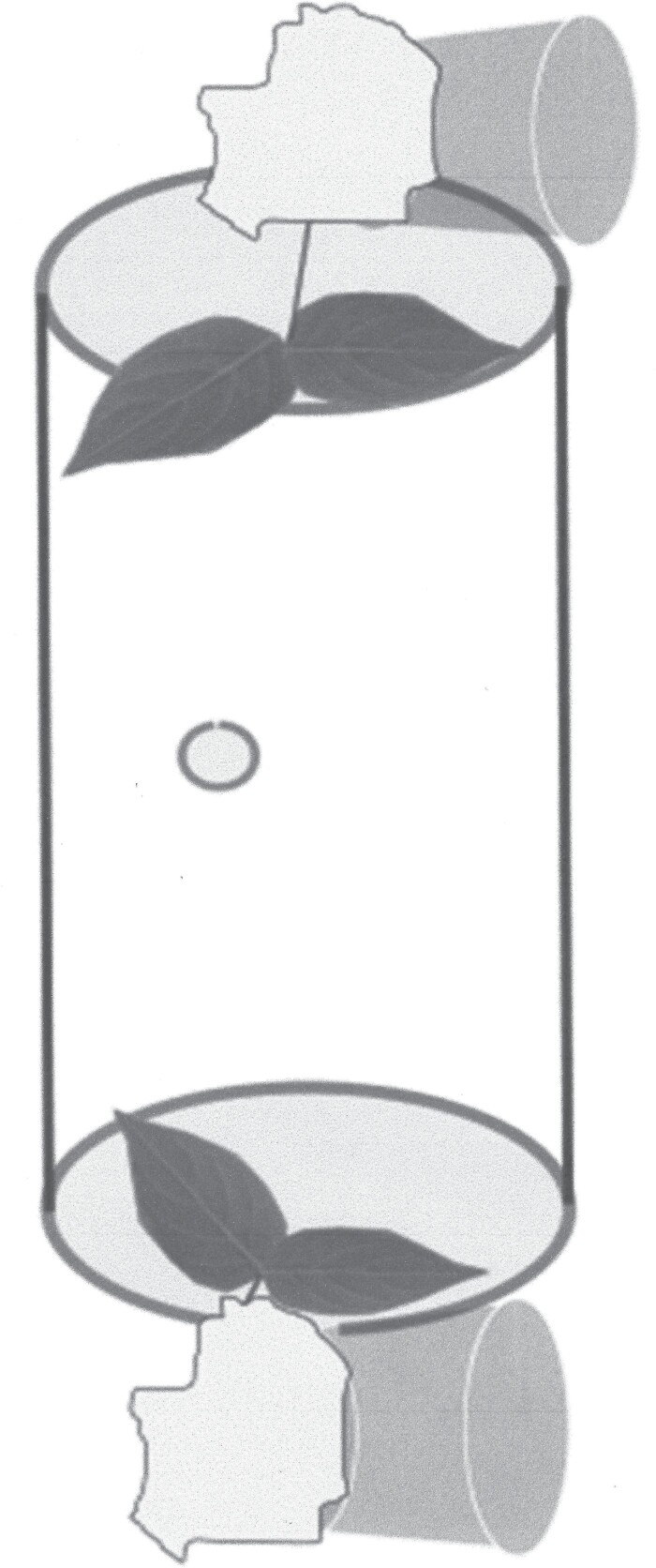
Laboratory-containerized choice assay design. Acrylic tube shown with hole drilled though one side of the tube in the center as well as mature and young hydrangea leaves at each end. Stems of the leaves were kept hydrated by moistened cotton balls resting on plastic cups.

Different sets of adults were collected from hydrangeas at the HRAREC on 19, 26, 27, 28, and 29 September 2022 and then starved for 24 h before starting these assays. Groupings of both 2–3 young and mature leaves were clipped from untreated ‘Limelight’ hydrangeas. A 14.5 cm length by 7.5 cm diameter acrylic tube had a 5-mm hole drilled through one side halfway down the length of it. Leaves with the stem attached and poked through a piece of parafilm were placed at the opposite ends of the tube (Fig. 1). Parafilm was secured to keep the leaves in place and seal the tube. The ends of the stems were surrounded by moistened cotton balls to prevent desiccation. One adult was placed into the tube using the drilled hole halfway between the leaves and then was sealed with another piece of parafilm. Leaf type was randomly assigned to each end of the tube. All replicates were placed in a growth chamber for 24 h with 26 °C, 55 ± 5% RH, and 14:10 L:D ratio. Acrylic tubes were placed in a freezer to kill the adults, so their sex could be determined. An individual tube was considered an experimental unit. For each experimental unit, the leaves were observed to determine whether injury was inflicted on either or both leaf types. Ten experimental units were run at a time. This trial was repeated 5 times, and the proportions of sex and leaf type preference were used as response variables to perform a linear mixed model (PROC MIXED; SAS). Fixed effects were sex of the adults, leaf type available, and the interaction between these 2 factors. The proportions of sex and leaf type preference were arcsine-root squared transformed for this analysis. Degrees of freedom calculations and mean separations post-ANOVA were conducted as previously mentioned.

## Results

### Individual Leaf Petri Dish Nonchoice Assays

#### Defoliation and area consumed collected from whole leaves

**Fig. 2.**
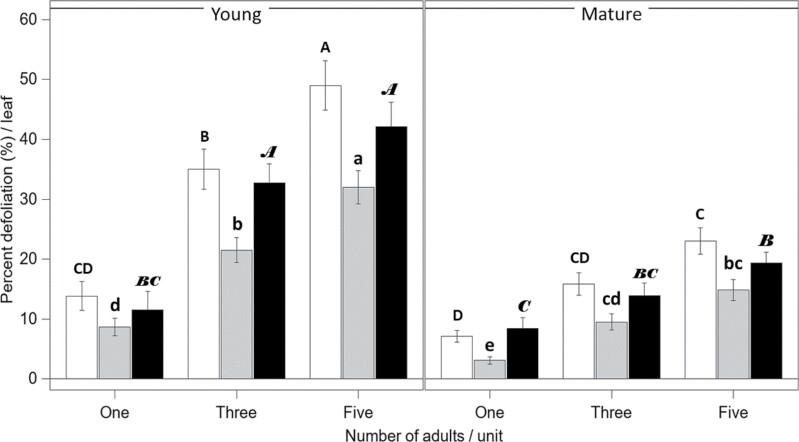
Percent defoliation ± SE originated by adult *Systena frontalis* and measured by Li-Cor area meter (white and black bars) and by the Leafbyte mobile application (gray bars). The x-axis represents the adult densities for each experimental unit. Individual vertical panels present defoliation values by each leaf tissue type (young vs. mature leaves). White and gray bars represent whole leaf assays, and black bars represent assays comparing cut-out mature leaf squares and young leaves. Uppercase and lowercase and italicized letters represent separate statistical analyses. Bars sharing the same letters are not statistically different (α = 0.05).

Percent defoliation of the leaves was influenced by the interaction between leaf type and number of adults (*F* = 4.09; df = 2, 161; *P* = 0.0185; Fig. 2). Additionally, time exposed had an impact on percent defoliation (*F* = 31.14; df = 1, 161; *P* < 0.0001), with higher defoliation recorded in 48 h. Leaf type, number of adults, and time exposed did not interact with percent defoliation of the leaves (*F* = 0.45; df = 2, 161; *P* = 0.64). There were no interactions between number of adults and time exposed (*F* = 1.44; df = 2, 161; *P* = 0.24), and leaf type and time exposed (*F* = 0.12; df = 1, 161; *P* = 0.73). Notably, 1 adult in 1 day consumed on average 10.88 ± 3.44 % of a young leaf and 4.61 ± 0.80 % of a mature leaf.

When LeafByte data were analyzed, there were no interactions influencing defoliation among leaf type, number of adults, and time exposed (*F* = 1.07; df = 2, 164; *P* = 0.35) or between number of adults and time exposed (*F* = 1.78; df = 2, 164; *P* = 0.17) and leaf type and time exposed (*F* = 0.05; df = 1, 164; *P* = 0.82). Leaf type interacted with the number of adults to affect the percent defoliation (*F* = 3.08; df = 2, 164; *P* = 0.0485; Fig. 2). Time exposed also affected the percent defoliation of a leaf (*F* = 43.77; df = 1, 164; *P* < 0.0001), where leaves exposed to adults for 48 h had higher percent defoliation.

Leaf type did not affect the area consumed (*F* = 0.38; df = 1, 160; *P* = 0.54). Both the number of adults (*F* = 77.53; df = 2, 161; *P* < 0.0001) and the time leaves were exposed (*F* = 71.34; df = 1, 161; *P* < 0.0001) significantly influenced the area consumed. Larger areas were consumed from leaves exposed to 5 adults (2.48 ± 0.15 cm^2^), followed by leaves with 3 adults (1.75 ± 0.12 cm^2^) and 1 adult (0.74 ± 0.08 cm^2^). Additionally, leaves enclosed with adults for 48 h had more area consumed (2.13 ± 0.13 cm^2^) when compared with leaves exposed for only 24 h (1.18 ± 0.09 cm^2^). No interactions were found between leaf type and number of adults (*F* = 1.03; df = 2, 160; *P* = 0.36), number of adults and time exposed (*F* = 1.3; df = 2, 161; *P* = 0.28), leaf type and time exposed (*F* = 0.09; df = 1, 160; *P* = 0.76), or among the 3 factors (*F* = 0.43; df = 2, 160; *P* = 0.65) with respect to leaf area consumed.

#### Defoliation and area consumed collected from young and square cut-out leaves

Percent defoliation for these sets of assays was also influenced by the interaction between leaf type and number of adults (*F* = 4.66; df = 2, 81; *P* = 0.0121; Fig. 2). Time exposed had an impact on percent defoliation (*F* = 20.03; df = 1, 81; *P* < 0.0001), with higher defoliation documented at 48 h. Leaf type, number of adults, and time exposed did not interact with percent defoliation of the leaves (*F* = 0.01; df = 2, 81; *P* = 0.99). There were no interactions between number of adults and time exposed (*F* = 2.19; df = 2, 81; *P* = 0.12), and leaf type and time exposed (*F* = 1.07; df = 1, 81; *P* = 0.31).

For the LeafByte data, there were no interactions influencing defoliation among leaf type, number of adults, and time exposed (*F* = 0.27; df = 2, 84; *P* = 0.77) or between number of adults and time exposed (*F* = 1.00; df = 2, 84; *P* = 0.37), leaf type and time exposed (*F* = 0.92; df = 1, 84; *P* = 0.34), and leaf type and the number of adults (*F* = 2.88; df = 2, 84; *P* = 0.06). Leaf type (*F* = 37.26; df = 1, 84; *P* < 0.0001), number of adults (*F* = 40.70; df = 2, 84; *P* < 0.0001), and time exposed (*F* = 13.26; df = 1, 84; *P* = 0.0005) affected the percent defoliation of leaves, where higher defoliation was documented from young leaves, or leaves with 5 adults, or leaves exposed to adults for 48 h.

Leaf type (*F* = 4.52; df = 1, 81; *P* = 0.0366), number of adults (*F* = 31.46; df = 2, 81; *P* < 0.0001), and the time leaves were exposed (*F* = 33.12; df = 1, 81; *P* < 0.0001) significantly influenced the area consumed. Young leaves had more area consumed (1.62 ± 0.16 cm^2^) when compared with the cut-out mature leaves (1.26 ± 0.12 cm^2^). Larger areas were consumed from leaves exposed to 5 adults (2.05 ± 0.16 cm^2^), followed by the ones with 3 adults (1.55 ± 0.16 cm^2^) and by leaves with one adult (0.72 ± 0.12 cm^2^). More leaf area was also consumed while tissue was exposed to adults for 48 h (1.84 ± 0.14 cm^2^), compared with leaves exposed for 24 h (1.05 ± 0.12 cm^2^). No interactions were found affecting the leaf area consumed between leaf type and number of adults (*F* = 1.90; df = 2, 81; *P* = 0.16), number of adults and time exposed (*F* = 2.6; df = 2, 81; *P* = 0.08), leaf type and time exposed (*F* = 2.07; df = 1, 81; *P* = 0.15), or among the 3 factors (*F* = 0.03; df = 2, 81; *P* = 0.97).

### Old Versus New Growth Whole-Plant Choice Assays

**Fig. 3.**
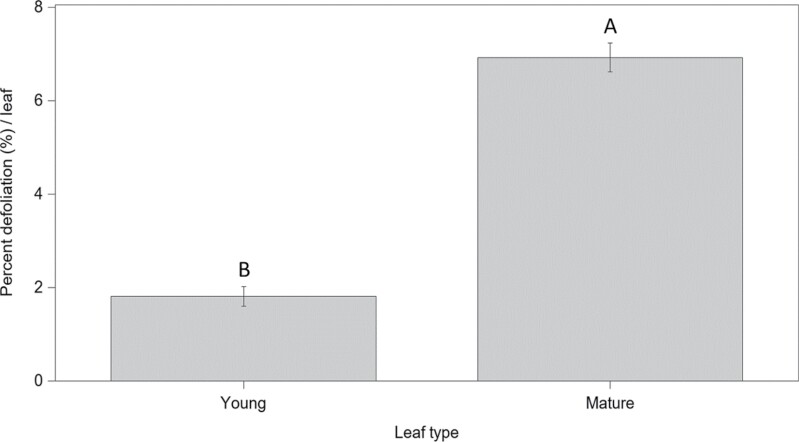
Percent defoliation ± SE to young and mature hydrangea leaves (x-axis) in caged choice assays using whole plants, resulted from adult *Systena frontalis* feeding. Bars with different letters indicate that means differ significantly (α = 0.05).

Percent defoliation of leaves was significantly different between leaf types (*F* = 158.81; df = 1, 18; *P* < 0.0001; Fig. 3). Similar trends were observed across repetitions (*F* = 1.53; df = 2, 18; *P* = 0.24), with no significant interaction between leaf type and repetitions (*F* = 0.57; df = 2, 18; *P* = 0.58). There was a preference for mature leaves that suffered higher percent defoliation when compared with young leaves (Fig. 3).

### Laboratory-Containerized Choice Assays

**Fig. 4.**
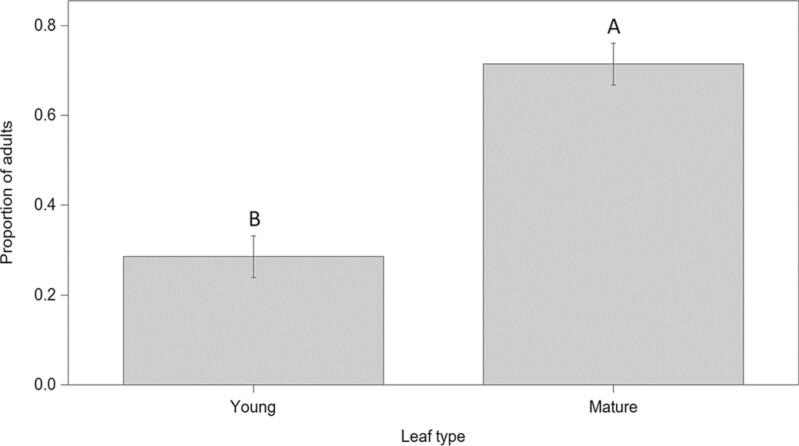
Proportion ± SE of adult *Systena frontalis* that fed on different hydrangea leaf types (x-axis), collected from laboratory-containerized choice assays. Bars with different letters indicate that means differ significantly (α = 0.05).

In laboratory-contained choice assays, adults showed a difference in which leaf type they preferred (*F* = 17.61; df = 1, 16; *P* = 0.0007; Fig. 4). Seventy percent of adults chose to feed on mature leaf clusters rather than young leaves. One beetle showed no preference and fed on both types of leaves. The proportion of females randomly selected for these assays was higher than males (*F* = 10.09; df = 1, 16; *P* = 0.0059). However, the interaction between sex and leaf type did not influence the proportion of adults recorded feeding on either young or mature leaves (*F* = 0.08; df = 1, 16; *P* = 0.79).

## Discussion

Virginia nurseries suffer economic losses caused by *S. frontalis* foliar feeding on ornamentals such as hydrangeas, sweetspire itea (*Itea virginica*, Iteaceae), and hollies (*Ilex* spp., Aquifoliaceae). To understand the feeding biology of this pest on hydrangeas, the injury that they inflict on mature and young leaves was evaluated. In the laboratory, nonchoice assays showed that individual young leaves had a higher percent defoliation than mature whole leaves. Once expanded to the whole-plant scale assays, mature leaves suffered a higher percent defoliation, contrasting the findings of the individual leaf assays. To document whether a preference was occurring, a 2-way containerized choice assay was conducted, which confirmed findings at the whole-plant scale. Mature leaves were repeatedly chosen over the young leaves within these choice assays. The corroboration of the 2-way choice assays and the whole-plant assays showed that *S. frontalis* fed more on mature leaves when given a choice. The discrepancy between the choice and nonchoice assays also showed that adults could feed on any available leaf tissue of a susceptible host.

In the individual leaf Petri dish assays, we expected to document a higher percent and amount of leaf area fed on in 48 h rather than 24 h, in young leaves rather than mature leaves, and in 5 beetles rather than 3 or 1 beetle. These hypotheses were found to be correct within the confines of this nonchoice experiment. Young leaves were more tender and injury was expected to be higher when compared with a mature leaf. Leaf area damaged was expected to be different between the leaf types since other studies have shown selection in favor of different leaf types, based on age. Specialist leaf beetles have been shown to selectively feed on young leaves in choice assays (Ernest 1989). Because *S. frontalis* is in the same family, a similar trend was expected. However, adult *S. frontalis* are polyphagous (Joseph et al. 2021); therefore, the opposite occurred as they followed the patterns of other polyphagous insects (Cates 1980). The polyphagous insects inflicted more injury on mature leaves because they were avoiding the more highly concentrated toxins within the younger leaves (Cates 1980). The expectation of *S. frontalis* choice of leaf type may be dependent on the host plant physiology, as woody perennials have higher levels of tannins and phenolic compounds in younger leaves compared with mature leaves (Feeny 1976). Therefore, it was hypothesized that hydrangeas would be similar and *S. frontalis* would follow the same pattern as the polyphagous insects and avoid feeding on the younger leaves.

Across all treatments and replications of these individual nonchoice leaf assays, only 2 individual leaves were almost 100% consumed. The injury to these leaves was a mixture of skeletonization and shot holes. Insects that skeletonize plant leaves feed on the mesophyll and leave the epidermis intact (Hochuli 2001). Severe skeletonization results in shriveled leaves without complete removal of plant tissue. In the field, growers would not scout for these severely shriveled leaves but instead for highly injured leaves (E.L.L., personal observation). Skipping injured leaves may result in underestimating the existing damage generated by this pest. When infestation levels were high and foliage was greatly consumed, adults were observed feeding on flower petals when infested ornamentals were left untreated (E.L.L., personal observation). As growers tend to prune away flowers under commercial nursery conditions, flower feeding would not be of consequence.

Whole leaves were used for the Petri dish nonchoice assays to maintain the similarity to field conditions, where adults are exposed to this type of tissue. Mature leaves were cut down to a smaller standardized size to more accurately compare the area damaged in young and mature leaves. These leaf artifacts were not representative of field conditions but created a more controlled assay, in terms of leaf area presented to adult *S. frontalis*. Cutting and exposing edges may have affected the distribution of any volatiles, but was necessary to elicit the response comparing the same leaf area prone to damage in selected leaf tissues. By using both area meter and mobile application, a more accurate conclusion can be made about the feeding potential of these adults on individual leaves. The resulting leaf area damaged from these standardized cut-out leaves was higher in young leaves when compared with mature ones. Both the higher percent damage and area consumed by younger leaves indicated a potential preference to be explored further.

To corroborate the previous findings from the nonchoice assays, a larger scale experiment was set up to document the feeding behavior of adults. Percent defoliation of mature or young leaves on the scale of an entire plant was observed and found to contradict data generated from the Petri dish individual nonchoice leaf studies. The adults inflicted higher percent defoliation on the mature leaves when given the choice on a caged whole plant. This corresponded with the hypothesis based on other polyphagous insect feeding behavior (Cates 1980). As a polyphagous insect, *S. frontalis* did not behave as other specific leaf beetles on feeding patterns at the plant level (Ernest 1989). An important distinction was the host plant difference in this study compared with others. The only study of feeding preference in *S. frontalis* was on cranberry foliage and showed more injury due to feeding closer to the apical ends (younger growth) (Jaffe et al. 2021).

Since there were contradictions among the Petri dish nonchoice assays with the choice caged plant assays, an additional experiment was set up to elucidate these opposite results. Two-way containerized choice assays found adults tend to feed on mature leaves more often than on young ones. This corroborated the findings of the whole-plant caged assays. Because these 2 experiments had the same conclusions, it could be hypothesized that adults will likely feed more often on mature leaves when given a choice and under field conditions. The findings of the Petri dish assays indicated that adults were able to consume a higher percentage of the younger leaf, potentially due to its smaller size when compared with whole mature leaves. In the 2-way choice assays, the mature leaves were trimmed to ensure adults had comparable amounts of leaf and distance to choose from. Even with this trimming, adults still fed on mature leaves. This result emphasized that physical damage on leaves might not deter *S. frontalis* adults from feeding on this type of tissue.

The combined findings from each experiment demonstrated that *S. frontalis* inflicted higher percent defoliation on mature leaves when given the choice, and likely under field conditions, but will feed and consume any type of leaves when restricted on their availability. This conclusion suggests *S. frontalis* preference toward mature leaves in hydrangeas. The mechanism behind these choices is still unclear, and the experiments in this study were not set up to address that question. Several factors could have influenced leaf tissue preference for these beetles, including plant host phenological stage, beetle reproductive status, and other environmental factors. It is also possible that secondary compounds may be more concentrated in younger leaves leading to deterrence of adults from this type of tissue. Further studies could elaborate on this behavior by identifying compounds from young and mature hydrangea leaves that might have some activity on adults. The adult responses to the different leaf types could be analyzed using an electroantennogram to determine whether chemosensing affects their choice with respect to leaf type. Although further investigation into causation is needed for this behavior, this study demonstrates a preference for mature leaves over young leaves in hydrangeas.
